# Supplementary material for: Influence of resonant plasmonic nanoparticles on optically accessing the valley degree of freedom in 2D semiconductors
Source: Nat Commun. 2024 Nov 21;15:10098. doi: 10.1038/s41467-024-54359-y (PMC11582587; doi:10.1038/s41467-024-54359-y)
Supplement: Supplementary file 1 — Supplementary Information [file 41467_2024_54359_MOESM1_ESM.pdf]

Supporting Information: Influence of resonant plasmonic nanoparticles on optically accessing the valley degree of freedom in 2D semiconductors

Tobias Bucher,[*,1,2,3,†] Zlata Fedorova,[*,1,2,3,†] Mostafa Abasifard,[2,1,3] Rajeshkumar Mupparapu,[2,3] Matthias J. Wurdack,[4,5,1,2,3] Emad Najafidehaghani,[6] Ziyang Gan,[6] Heiko Knopf,[7,2,3,8] Antony George,[6,3] Falk Eilenberger,[7,2,3,8] Thomas Pertsch,[2,3,7,8] Andrey Turchanin,[6,3,9] and Isabelle Staude[1,2,3,8]

1*Institute of Solid State Physics, Friedrich Schiller University Jena, 07743 Jena, Germany*

2*Institute of Applied Physics, Friedrich Schiller University Jena, 07745 Jena, Germany*

3*Abbe Center of Photonics, Friedrich Schiller University Jena, 07745 Jena, Germany*

4*Department of Chemical Engineering, Stanford University, Stanford, CA, USA*

5*ARC Centre of Excellence in Future Low-Energy Electronics Technologies and Department of Quantum Science and Technology, Research School of Physics, The Australian National University, Canberra, ACT, 2601, Australia*

6*Institute of Physical Chemistry, Friedrich Schiller University Jena, 07743 Jena, Germany*

7*Fraunhofer Institute for Applied Optics and Precision Engineering IOF, 07745 Jena, Germany*

8*Max Planck School of Photonics, Germany*

9*Jena Center for Soft Matter (JCSM), 07743 Jena, Germany*

†*These authors contributed equally*

E-mail: tobias.bucher@uni-jena.de; zlata.fedorova@uni-jena.de

S.1 Influence of the optical components on the detected polarization state of the PL

Cryogenic photoluminescence (PL) microscopy experiments were performed in reflection mode, as depicted in Figure S1a. In this setup, the incoming laser beam initially is passed through a linear polarizer (LP, Thorlabs LPVISC050-MP2) and a quarter-wave plate (QWP, Thorlabs WPMQ05M-633), then is reflected by a non-polarizing 30(R):70(T) plate beam splitter (BS, Chroma) and subsequently is focused onto the sample via an objective lens (Zeiss 422392-9900-000, EC Epiplan-Neofluar 100x/0.90 DIC Vak objective) inside the vacuum chamber of the cryostat. The circular polarization state of the laser is routinely evaluated immediately after the BS, at a point marked as position 1 in Figure S1. The PL emitted from the sample is recollected by the same objective, transmitted through the BS, and then redirected by a silver mirror (Thorlabs PF10-03-P01). Subsequently, the PL is analysed by a superachromatic QWP (SAQWP, Thorlabs SAQWP05M-700) and a LP (Thorlabs LPVIS100-MP2). This arrangement was motivated by the specific design of the cryostat chamber and the restrictions of the laboratory space. We will further investigate the influence of the BS and the mirror on the measured degree of circular polarization (DOCP) of the PL in such a setup.

S.1.1 Beam splitter

First, we have analyzed the BS in a custom-build white-light spectroscopy setup for near-zeroth order transmittance (NA$\approx$ 0.044) and under 45° incidence angle. In this setup, the incoming white light is prepared in an arbitrary polarization state, transmitted through the BS, and fiber-coupled into a spectrometer. By adding a rotatable QWP and fixed LP before the spectrometer fiber, we are able to measure the components of the Stokes vector (Fourier method) of the transmitted light. Initially, we characterized the transmitted light for six degenerate input polarization states, namely the four linear polarized states vertical

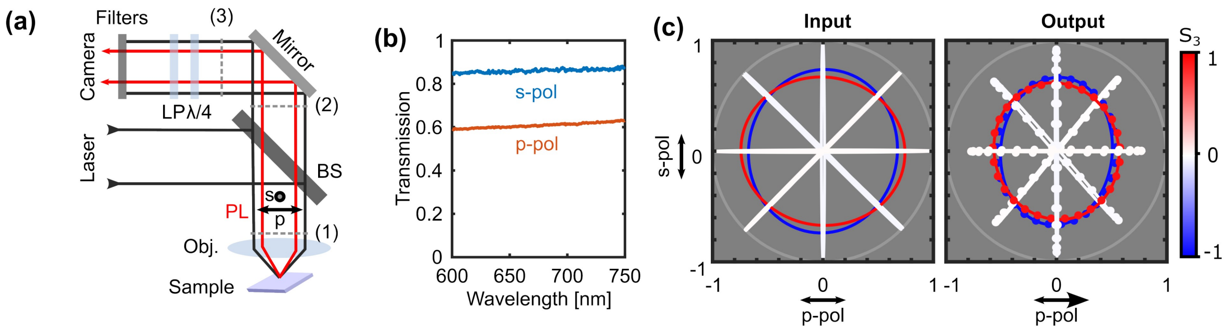

Fig. S1: Stokes polarimetry in the cryostat setup. (a) Sketch of the optical setup in reflection mode. The numbers in round brackets next to the gray dashed lines denote different positions. (b) Intensity transmission of s- and p-polarized light over a broad spectral range. (c) Polarization ellipses of the 6 analyzed cases averaged over the wavelength range from 650 nm up to 670 nm. Here, "Input" refers to the polarization state measured without a BS while "Output" refers to the polarization state after passing the BS. The circles in the "Output" plot show the theoretically calculated ellipses based on the estimated Müller matrix and the input state. The line color encodes the corresponding DOCP for clear differentiation between left- and right-handed circularly polarized light.

(s-polarization), horizontal (p-polarization), diagonal and anti-diagonal and for left- and right-handed circular polarization. The respective polarization ellipses measured for the "Input" (without BS) and "Output" (with BS) are shown in Figure S1c, respectively. By comparison, we mainly observe three aspects: (1) the polarization ellipses are squeezed with respect to the horizontal axis (p-polarization), (2) the ellipticity of linearly polarized input states does not change and (3) the polarization ellipses for circularly polarized input states are not rotated. To describe the polarization behaviour of the BS we employ the Müller matrix formalism, where the output Stokes vector is related to the input Stokes vector via $\mathbf{S}^{\text{out}} = M_{BS}\mathbf{S}^{in}$. Based on the aforementioned observations, we conclude that our BS acts as a linear polarizing element with anisotropic amplitude attenuation coefficients p_s and p_p. Thus, the corresponding Müller matrix should have the form[1]

$$M_{BS} = \frac{1}{2}\begin{bmatrix} p_s^2 + p_p^2 & p_s^2 - p_p^2 & 0 & 0 \\ p_s^2 - p_p^2 & p_s^2 + p_p^2 & 0 & 0 \\ 0 & 0 & 2p_sp_p & 0 \\ 0 & 0 & 0 & 2p_sp_p \end{bmatrix}. \tag{1}$$

Here, p_s^2 and p_p^2 are intensity transmittances, $p_s^2 = T_s$ and $p_p^2 = T_p$, which are shown in Figure S1b for a wide spectral range from 600 nm to 750 nm. The transmission characteristics of the BS exhibited minimal dependence on wavelength, with average transmission coefficients for the intensity of T_s =0.86 and T_p =0.61 for s- and p-polarized light, respectively. Based on these values, the corresponding Müller matrix is calculated in a straightforward manner. In Figure S1c we observe a strong agreement between the measured output polarization ellipses (solid lines) and the transformed input ellipses (circles) using computed M_{BS}. To understand how the BS influences the detected DOCP of the PL, let us consider a partially circularly polarized light as an input, i.e. $\mathbf{S}^{\text{in}} = [1, 0, 0, \alpha]^{\text{T}}$ where $\alpha \in (-1, 1)$ is a degree of circular polarization (DOCP). After the BS at the position 2 in Figure S1a we obtain: $\mathbf{S}^{\text{out}} = M_{BS}\mathbf{S}^{in} = 0.735 \cdot [1, 0.170, 0, 0.985 \cdot \alpha]^{\text{T}}$. Thus, the BS induces the constant positive offset of S_1, S_2 remains zero, while the DOCP, represented by the S_3, is only negligibly decreased by a factor of 0.985 (when normalized by S_0).

S.1.2 Mirror

After the BS, the PL is reflected from a protected silver mirror under 45°. The Müller matrix for reflection from metals can be given in terms of the reflection coefficients r_s and r_p [2]

$$M_{\text{Mirror}} = \frac{1}{2}\begin{bmatrix} r_s^2 + r_p^2 & r_s^2 - r_p^2 & 0 & 0 \\ r_s^2 - r_p^2 & r_s^2 + r_p^2 & 0 & 0 \\ 0 & 0 & 2r_s r_p \cos\gamma & -2r_s r_p \sin\gamma \\ 0 & 0 & 2r_s r_p \sin\gamma & 2r_s r_p \cos\gamma \end{bmatrix}, \tag{2}$$

where $\gamma = \gamma_s - \gamma_p$ is the phase offset between s- and p- polarized field components. According to the manufacturer, the reflectance of the mirror at the PL wavelength and for 45° incidence angle are $r_s^2 = R_s \approx 0.97$ and $r_p^2 = R_p \approx 0.95$. Considering the normalized Stokes vector after the BS as $\mathbf{S}^{in} = [1, 0.17, 0, 0.985 \cdot \alpha]^{\text{T}}$, it will be transformed by the mirror as follows $\mathbf{S}^{\text{out}} = M_{\text{Mirror}}\mathbf{S}^{in} = 0.962 \cdot [1, 0.18, -0.983 \cdot \alpha \sin\gamma, 0.983 \cdot \alpha \cos\gamma]^{\text{T}}$. This analysis predicts that the

detected polarization state of the PL will be influenced by both optical elements. Specifically, S_1 acquires a positive offset that is independent of the DOCP, S_2 shifts proportionally to the DOCP, and crucially, S_3 remains directly proportional to the DOCP.

S.1.3 Stokes polarimetry for the laser beam

To validate our analysis we assessed the polarization state of the laser prepared in σ_+/σ_- state at the positions numbered as 1 and 3 in Figure S1a. The normalized Stokes components $\mathbf{S} = (1, S_1, S_2, S_3)$ were defined as follows:

$$S_1 = \frac{\mathcal{I}_s - \mathcal{I}_p}{\mathcal{I}_s + \mathcal{I}_p}, \quad S_2 = \frac{\mathcal{I}_d - \mathcal{I}_a}{\mathcal{I}_d + \mathcal{I}_a}, \quad S_3 = \frac{\mathcal{I}_{\sigma^+} - \mathcal{I}_{\sigma^-}}{\mathcal{I}_{\sigma^+} + \mathcal{I}_{\sigma^-}}, \tag{3}$$

where $\mathcal{I}$ denotes the light intensity, while the subscript indicates the detection polarization: linear (s) or (p), linear diagonal (d)/ antidiagonal (a), as well as two circular polarizations σ_+/σ_-. The obtained results are summarized in the table below:

**S**	σ^+, Position 1	σ^+, Position 3	σ^-, Position 1	σ^-, Position 3
S1	0.003 ± 0.003	0.256 ± 0.006	-0.008 ± 0.003	0.173 ± 0.004
S2	0.006 ± 0.003	0.142 ± 0.005	0.01 ± 0.003	-0.178 ± 0.004
S3	0.977 ± 0.003	0.839 ± 0.003	-0.972 ± 0.005	-0.8673 ± 0.002

These measurements agree well with our expectations. Namely, the laser, initially prepared in an almost perfect σ^+ (or σ^-) polarization state at position 1, undergoes changes due to the BS and the mirror. This results in S_1 becoming positive, close to 0.18 as anticipated, S_2 shifting in proportion to the degree of circular polarization (DOCP), and S_3 showing a slight reduction. We attribute the minor discrepancies from the expected outcomes to misalignments of the BS and the mirror, as well as to unaccounted polarization effects from the objective lens.

S.2 Polarization-resolved cryo-PL microscopy

We studied the modification of the farfield DOCP of valley-specific emission from monolayer molybdenum disulphide (1L-MoS_2) by resonant gold nanoparticles (GNP). In order to infer changes in the degree of polarization from the DOCP alone, linear polarization components must vanish identically. Hence, we have performed full Stokes-polarimetric measurements of the photoluminescence (PL) from 1L-MoS_2 decorated with GNPs. Figure S2a shows the

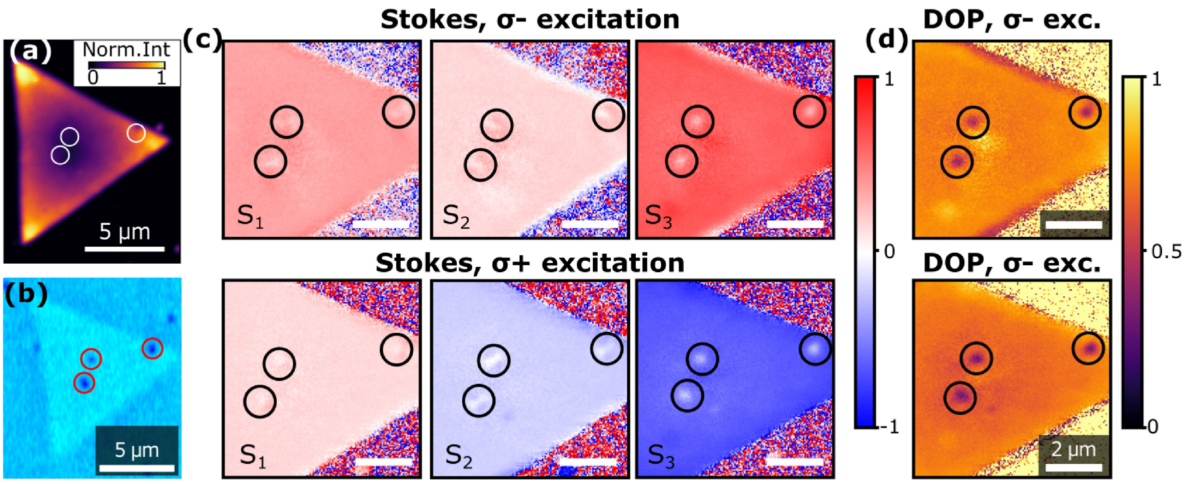

Fig. S2: Polarization resolved photoluminescence imaging. (a) Measured confocal scan of the total emission intensity $\mathcal{I}_{\text{tot}}$ from embedded 1L-MoS_2 decorated with monodispersed GNPs upon σ^+ excitation and collected through a 660 nm bandpass filter. (b) Optical microscopy image of the same sample. In both images the position of the GNPs is indicated by circles. (c) Measured confocal scans of the Stokes parameters of emission from the same sample upon σ^- (top row) and σ^+ (bottom row) excitation. (d) Corresponding degree of polarization (DOP) for two excitation polarizations. All scale bars in (c) and (d) represent 2 µm.

measured total PL intensity $\mathcal{I}_{\text{tot}} = (\mathcal{I}_{\sigma^+} + \mathcal{I}_{\sigma^-})$ at 660 nm wavelength of a single crystal of 1L-MoS_2 decorated by GNPs. Figure S2b shows an optical microscopy image of the same sample and in both cases the GNPs are marked by circles, respectively. The GNPs have a negligible effect on the total emission intensity of 1L-MoS_2 as no significant variation in brightness can be associated with the positions of the GNPs. In contrast, when calculating the respective Stokes parameter scans, as shown in Figure S2c for σ^- (top row) and σ^+ (bottom row) polarized excitation, we find a clear modulation of the polarization properties

of emission from 1L-MoS_2 mediated by the GNPs. For the DOCP or S_3 (right column), we find again a strong reduction as described in the main text. Furthermore, we observe the offsets of S_1 and S_2 induced by the utilized optical components as detailed in the previous section. These offsets align well with the behaviour of the initially circularly polarized laser light that passes through the same optical components (see subsection S.1.3). At the center of the GNPs, S_2 approaches nearly zero, affected by the DOCP of the circularly polarized PL due to mixing of the Stokes vector components by the mirror. Around the nanoparticles, we observe subtle lobes of enhanced S_1 and S_2 indicating that it induces a mild linear polarization in its vicinity. Nonetheless, complete depolarization is evident at the center, as depicted in Figure S2d, where the degree of polarization is calculated using the formula:

$$\mathrm{DOP} \equiv \sqrt{S_1^2 + S_2^2 + S_3^3}. \tag{4}$$

This calculation reveals that the minor increase in linear polarization around the nanoparticle has a negligible impact on the overall degree of polarization, which is primarily affected by the decrease in S_3 Therefore, the alteration in S_3 is directly linked to changes in the degree of polarization.

S.3 Polarization properties of valley-selective excitonic emitters

For modelling emitters of circularly polarized light mainly two dipolar emitter types are discussed in literature:[3–5] (i) the rotating electric dipole $\vec{\mathbf{p}}_{K/K'} = \vec{\mathbf{p}}_x \pm i\vec{\mathbf{p}}_y$ where the spin-angular momentum of the emitter determines the sense of rotation and (ii) the chiral dipole $\vec{\mathbf{p}}_{\sigma^\pm} = \vec{\mathbf{p}}_x \pm i\vec{\mathbf{m}}_x$ whose emission has a defined helicity due to duality invariance. In the context of 1L-TMDs, strong spin-orbit coupling as well as a broken inversion symmetry lead to valley-contrasting selection rules, i.e. the circularly-polarized external field $\sigma^\pm$ excites

valley-polarized excitons, commonly approximated by rotating electric dipoles. The inset of

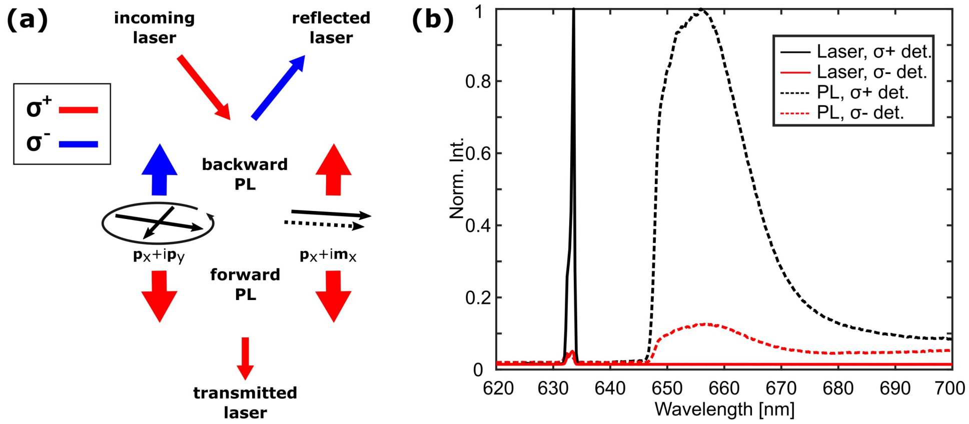

Fig. S3: Comparison of the polarization contrast of reflected laser and emission. (a) Sketch indicating the circular polarization of excitation light and emitted PL for the case of a rotating electric dipole (left side) and a chiral dipole (right side) upon σ^+ polarized excitation. (b) Normalized spectra of the σ^+-polarized HeNe laser detected after the reflection from the sample (solid lines) and of the photoluminescence from the bare 1L-MoS_2 excited by this laser (dashed) for σ_+ (black) and σ_- (red) detection polarizations.

Figure 5 (a) of the main text shows the farfield intensity distribution and respective DOCP of a single counterclockwise rotating electric dipole. Such a rotating dipole emits circularly polarized light with opposite helicities into different half spaces with an average of exactly zero (as opposed to a chiral dipole whose helicity averages to ± 1).

Here, we want to confirm experimentally that the polarization of the PL from a bare 1L-MoS_2 qualitatively follows the behaviour of a rotating electric dipole. In reflection geometry and given a fixed excitation polarization, we can compare the polarization state of the valley-selective PL emitted in backwards direction to the that of the reflected laser beam as sketched in Figure S3a for a σ^+ polarized excitation. The rotating dipole (left side) inherits the spin-angular momentum from the excitation field and therefore emits light with the same handedness as the incoming laser beam into forward direction but with opposite handedness into backward direction. Note that upon the reflection from the sample, the circular polarization of the incoming laser also flips, i.e. $\sigma^+ \longrightarrow \sigma^-$. Hence, the reflected laser light and the backwards emitted PL from a rotating dipole will be detected with the same circular polarization. Conversely, for the chiral dipole (right side) they would be detected

with equal handedness as the emission polarization state does not depend on the emission direction. Figure S3b shows the measured polarization-resolved spectra of backwards emitted PL and reflected laser light from bare 1L-MoS_2 upon excitation by a σ^+ polarized laser beam (HeNe, 633 nm). Both sets of spectra show a clear circular polarization contrast with each set of spectra favoring σ^+ polarization in detection. From this we can conclude that the polarization behaviour of bare 1L-MoS_2 is qualitatively matching that of a rotating electric dipole. Note that the reflected laser light and the backwards emitted PL should appear with σ^- polarization (as we have chosen σ^+ polarization for the excitation). However, before we detect it, the laser experiences an additional reflection from the mirror $\sigma^- \longrightarrow \sigma^+$ as shown in Figure S1a, position (3). As, a result, the excitation and detection polarization become the same.

S.4 Dependence of PL depolarization on the spacer thickness

To explore the generality of a GNP's effect on the PL polarization we fabricated samples with different thicknesses of the SiO_x spacer layer, namely 5 nm and 50 nm while maintaining identical conditions for other fabrication steps as described in the Methods section. Figure S4 (a-c) shows the result of confocal scanning microscopy of these two samples together with the case of 15 nm spacer from the main text for comparison.

We initially observe that the spacer thickness significantly affects the total PL intensity (see Figure S4 (d), left). Specifically, the PL intensity is enhanced at the nanoparticle's position with a 5 nm spacer, remains nearly unchanged with a 15 nm spacer, and decreases with a 50 nm spacer. This variation in intensity can be explained by the interplay between GNP's absorption and nearfield enhancement, which counteract each other at shorter distances. Despite variations in spacer thickness, all samples exhibit depolarization of the PL at the GNP location. Notably, the size of the depolarized (white) spot increases as the spacer

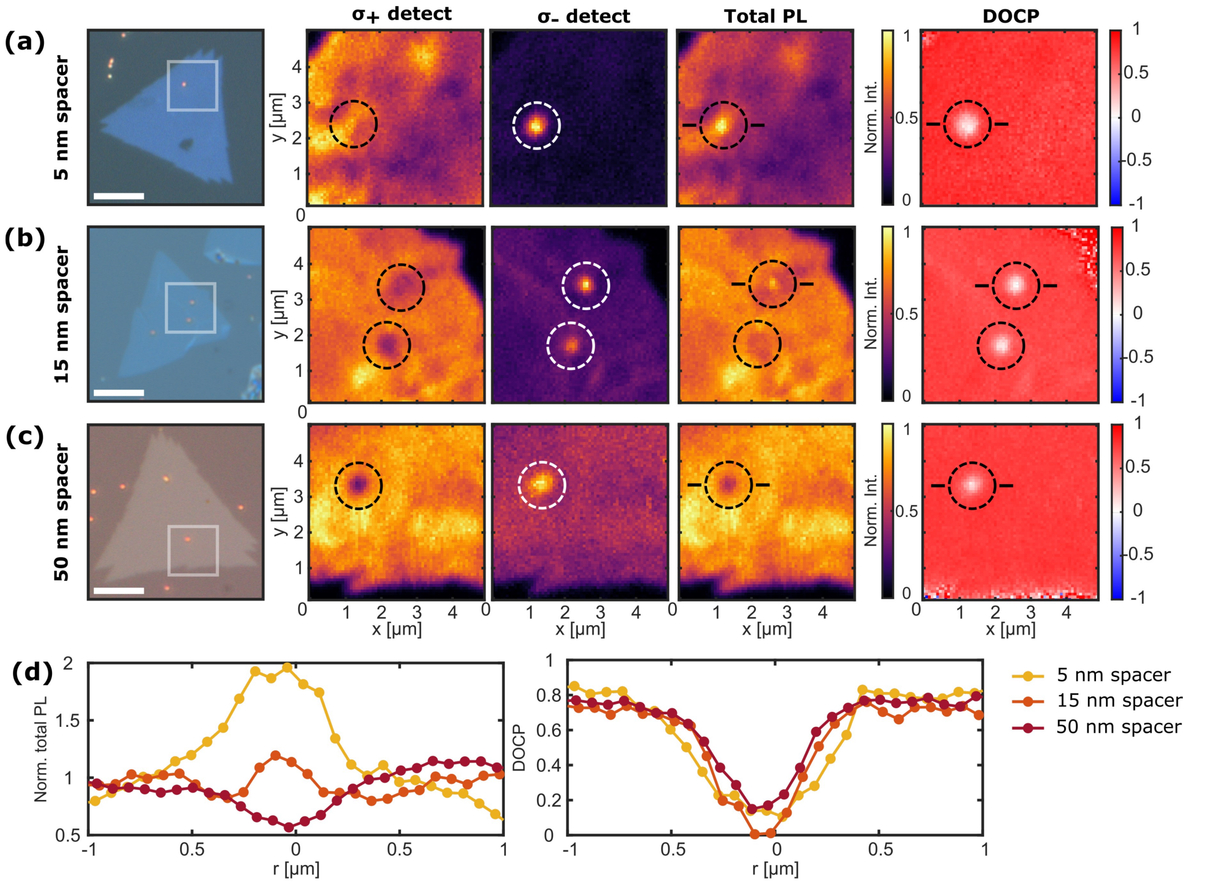

Fig. S4: Influence of the spacer thickness. (a-c) From left to right: Microscope images of the samples with highlighted scanning regions denoted by white squares, confocal scans showing the PL intensities for σ^+ and σ^- excitation, the total PL intensity, and the DOCP obtained for varying spacer thicknesses (5 nm, 15 nm, and 50 nm). The positions of the GNPs are indicated with circles, and short horizontal lines adjacent to these circles mark the locations where the cross-sections depicted in (d) are taken. (d) Cross-sections through the center of the GNPs showing total PL intensity (left) and the DOCP for the samples illustrated in (a-c).

thickness decreases (see Figure S4 (d), right). This effect is attributed to the narrowing of the numerical aperture (NA) of the light cone interacting with the GNP as the distance from the source to the GNP increases.

S.5 Nearfield excitation intensity distribution for a GNP

Figure S5 illustrates the formation of the intensity pattern below a GNP upon scattering of the tightly focused Gaussian beam incident from $z = +\infty$. Here, we only considered the field components that are parallel to the substrate surface, i.e. E_x and E_y. A ring-like intensity pattern forms near the GNP at distances less than 50 nm, while at larger distances, the intensity distribution resembles that of a Gaussian beam. All simulation parameters are identical to those described in the main text.

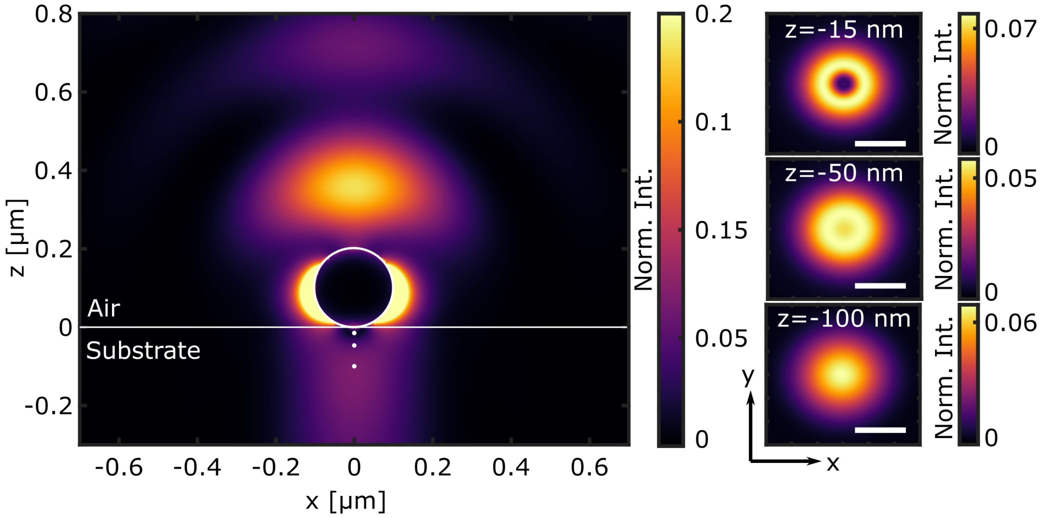

Fig. S5: Nearfield intensity distribution of a gold nanoparticle on substrate. Left: Numerically computed in-plane intensity $|E_x|^2 + |E_y|^2$ of a GNP upon scattering of a tightly focused Gaussian beam shown in a x-z plane intersecting the center of a GNP. The white dots below the GNP correspond to $z = -15$ nm, $z = -50$ nm, and $z = -100$ nm. Right: in-plane intensity $|E_x|^2 + |E_y|^2$ shown in x-y planes below the GNP at different z values. The scale bar corresponds to 200 nm.

S.6 Emergence of cross-polarized nearfields below a GNP

In Figure S6a we plot the intensities of the σ^+ and σ^- polarized in-plane field components in the x-y plane at $z = -15$ nm. We compare the cases of a focused Gaussian beam and a plane wave incident on a GNP, as well as the case of a focused Gaussian beam without a GNP as a reference. The excitation field is always σ^+ polarized while other simulation parameters are

kept identical to those described in the main text. In Figure S6b we analyze the resultant 2D-DOCP. Note that in the presence of a GNP and under Gaussian-beam excitation, a ring of reversed 2D-DOCP is observed. The location of this ring is easily deducible from Figure 4 in the main text, where the curves $I^{||}_{\sigma-}(r)$ and $I^{||}_{\sigma+}(r)$ cross. Conversely, for a plane wave, the curves do not intersect and the 2D-DOCP always stays positive with a slight reduction in the vicinity of the GNP. Reference data without a GNP confirms that the cross-polarized in-plane fields, leading to reduction or even reversal of 2D-DOCP, emerge only in the presence of a GNP.

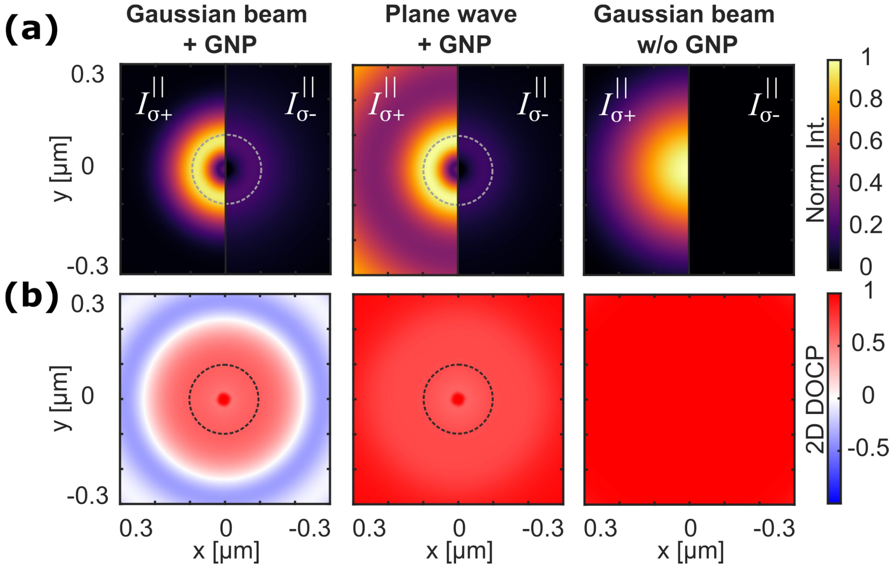

Fig. S6: Helical intensity components in the nearfield of a gold nanoparticle. (a) Numerically calculated $I^{||}_{\sigma+}(x, y)$ and $I^{||}_{\sigma-}(x, y)$ in the plane of the monolayer for different excitation scenarios. Only halves of the intensities are shown due to symmetry and both sides are normalized by the same value. The left column corresponds to a focused Gaussian beam on a GNP, the middle column - to the case of a plane wave incident on a GNP, and right column - to the focused Gaussian beam in the absence of a GNP. (b) Resultant 2D-DOCP in x-y plane. The dotted circles highlight the projected GNP edge.

S.7 Influence of a GNP on the total degree of polarization

In Sec. S2, we demonstrated that the reduction of DOCP caused by a GNP is accompanied by a decrease in the total degree of polarization. Here, we will numerically explore

this effect to assess whether our theoretical model accurately predicts this behaviour. The critical step in determining the expected DOP involves calculating the spatially-dependent Stokes parameters S_1 and S_2 as outlined in equation (4). Here, we focus on the scanning

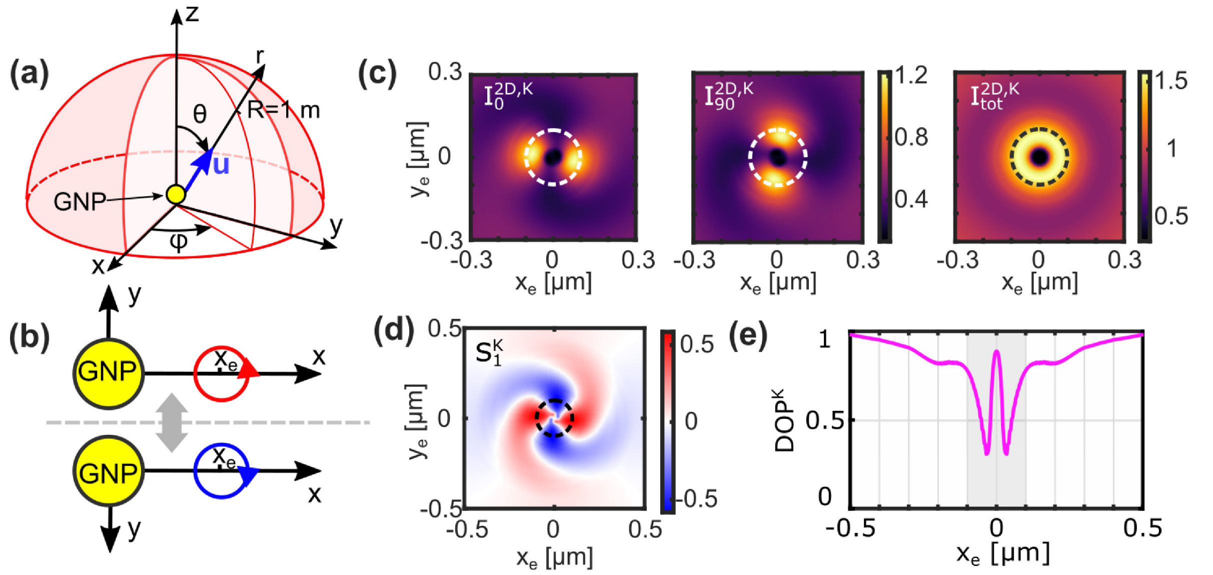

Fig. S7: Linear polrization properties of incoherent σ^+-rotating dipoles coupled to a GNP (a) Hemispherical surface with a radius of 1 m centered at the GNP (not in scale) on which the farfields are computed. The sketch shows the relation between the used Cartesian $\{x, y, z\}$ and spherical coordinate systems $\{\theta, \phi, r\}$ as well as the direction unit vector $\mathbf{u}$. (b) Sketch of the system's geometry showing that K and K' emitters are related by reflection around the symmetry axis (here, x-axis). (c) Calculated farfield intensities $\mathcal{I}_0^{2\mathrm{D},K}$ and $\mathcal{I}_{90}^{2\mathrm{D},K}$ and their sum $\mathcal{I}_{\mathrm{tot}}^{2\mathrm{D},K}$. The dotted circle denotes the projected GNP edge. (d) Spatially-dependent S_1^K. (e) Radial dependence of the DOP for homogeneously distributed incoherent K-emitters. The shaded region corresponds to the GNP size.

measurement scheme, using the same assumptions and settings detailed in the methods section. Experimentally, S_1 and S_2 are obtained by measuring light intensities through a linear polarizer rotated at 0°, 90°, 45°, and 135° degrees in the laboratory coordinate system (see equation (3)). To extract S_1 and S_2 we project the computed farfields given in spherical coordinates onto the fixed polarizer direction. These calculations rely on our FDTD simulations for a single σ^+ rotating dipole (K-valley) located on the xy-plane, $z = -15$ nm displaced along the x-axis by the distance x_e from the projected GNP's symmetry center (see Fig. 5 a). The corresponding farfield components on a hemispherical surface with a radius of R=1 m

centered around the GNP are $\{E_\theta^K(x_\mathrm{e},\mathbf{u}), E_\phi^K(x_\mathrm{e},\mathbf{u}), E_r^K(x_\mathrm{e},\mathbf{u})\}$, where K is the valley index and $\mathbf{u} = \mathbf{u}(\theta,\phi)$ denotes the emission direction in terms of a direction unit vector in spherical coordinates (see Figure S7a). As explained in the Methods section of the main text, we restrict ourselves to the finite numerical aperture of the objective ($\sin\theta < 0.9$). Note, that there is no need in repeating simulations for σ^- dipoles (K'-valley), since the corresponding farfields, up to a constant phase factor, can be retrieved by reflecting the distributions for K-emitters about the symmetry axis: $E_\theta^{K'}(x_\mathrm{e},\mathbf{u}(\theta,\phi)) = E_\theta^K(x_\mathrm{e},\mathbf{u}(\theta,-\phi))$ and $E_\phi^{K'}(x_\mathrm{e},\mathbf{u}(\theta,\phi)) = -E_\phi^K(x_\mathrm{e},\mathbf{u}(\theta,-\phi))$ (see Figure S7b). Electric field projections onto the polarizers at 0° and 90° with respect to x-axis and emitted into the direction $\mathbf{u}$ are given by

$$
\begin{aligned}
E_0^K(x_\mathrm{e},\mathbf{u}) &= E_\theta^K(x_\mathrm{e},\mathbf{u})\cos\phi - E_\phi^K(x_\mathrm{e},\mathbf{u})\sin\phi,\\
E_{90}^K(x_\mathrm{e},\mathbf{u}) &= E_\theta^K(x_\mathrm{e},\mathbf{u})\sin\phi + E_\phi^K(x_\mathrm{e},\mathbf{u})\cos\phi,
\end{aligned}
\tag{5}
$$

where ϕ is the azimuthal angle of the emission direction (see Figure S7a).

Next, we consider an arbitrary emitter position $(x_\mathrm{e}, y_\mathrm{e})$ on the xy plane. Due to the cylindrical symmetry of a GNP, the corresponding farfields can be directly obtained from our previous simulations for the displacement along the x-axis ~~$\mathbf{r} : r = \tilde{r}$~~ by rotating the coordinate system at an angle $\phi_\mathrm{e} = \mathrm{atan2}\,(y_\mathrm{e}, x_\mathrm{e})$, with $\mathrm{atan2}(y,x)$ denoting the four-quadrant inverse tangent of $\frac{y}{x}$. The sought projections of the electric fields emitted into the direction $\mathbf{u}$ are expressed as

$$
\begin{aligned}
E_0^{\mathrm{2D},K}(x_\mathrm{e},y_\mathrm{e},\mathbf{u}) &= E_\theta^K(\sqrt{x_\mathrm{e}^2+y_\mathrm{e}^2},\mathbf{u})\cos(\phi+\phi_\mathrm{e}) - E_\phi^K(\sqrt{x_\mathrm{e}^2+y_\mathrm{e}^2},\mathbf{u})\sin(\phi+\phi_\mathrm{e})\\
E_{90}^{\mathrm{2D},K}(x_\mathrm{e},y_\mathrm{e},\mathbf{u}) &= E_\theta^K(\sqrt{x_\mathrm{e}^2+y_\mathrm{e}^2},\mathbf{u})\sin(\phi+\phi_\mathrm{e}) + E_\phi^K(\sqrt{x_\mathrm{e}^2+y_\mathrm{e}^2},\mathbf{u})\cos(\phi+\phi_\mathrm{e}),
\end{aligned}
\tag{6}
$$

where by superscript '2D' we highlight the dependency of some quantity on 2D emitter distribution. The intensities through the 0°- and 90°-rotated polarizers from incoherent rotating dipoles integrated over all emission angles accepted by our objective's NA yields

$$
\mathcal{I}_{0/90}^{\mathrm{2D},K}(x_\mathrm{e},y_\mathrm{e}) \propto \iint |E_{0/90}^{\mathrm{2D},K}(x_\mathrm{e},y_\mathrm{e},\mathbf{u}(\theta,\phi))|^2 \sin\theta \mathrm{d}\theta\mathrm{d}\phi \tag{7}
$$

The distributions $\mathcal{I}_0^{2\mathrm{D},K}(x_\mathrm{e}, y_\mathrm{e})$ and $\mathcal{I}_{90}^{2\mathrm{D},K}(x_\mathrm{e}, y_\mathrm{e})$ normalized to the total intensity without a GNP are plotted in Figure S7c. We verify that their sum, $\mathcal{I}_{\mathrm{tot}}^{2\mathrm{D},K} = \mathcal{I}_0^{2\mathrm{D},K} + \mathcal{I}_{90}^{2\mathrm{D},K}$, restores the ring-like pattern and coincides with the result for the circular basis (main text, Fig. 5b). The resulting $S_1^K = (\mathcal{I}_0^{2\mathrm{D},K} - \mathcal{I}_{90}^{2\mathrm{D},K})/(\mathcal{I}_0^{2\mathrm{D},K} + \mathcal{I}_{90}^{2\mathrm{D},K})$ (see Figure S7d) forms spiraling lobes of opposite signs, reaching a maximum absolute value of 0.59 at the GNP's edge and decaying to zero further away. Similarly, the field projections onto polarizers at 45° and 135° are computed using equations (6) by shifting $\phi \longrightarrow \phi + \pi/4$ and substituting the indices $0 \longrightarrow 45$ and $90 \longrightarrow 135$. The intensities $\mathcal{I}_{45}^{2\mathrm{D},K}$ and $\mathcal{I}_{135}^{2\mathrm{D},K}$ are obtained with a counterpart of equation (7) with the replaced indices yielding $S_2^K = (\mathcal{I}_{45}^{2\mathrm{D},K} - \mathcal{I}_{135}^{2\mathrm{D},K})/(\mathcal{I}_{45}^{2\mathrm{D},K} + \mathcal{I}_{135}^{2\mathrm{D},K})$. The degree of polarization for homogeneously distributed incoherent K-emitters is then calculated as $\mathrm{DOP}^K = \sqrt{\sum_{i=1}^{3}(S_i^K)^2}$. The result in Figure S7e shows that the DOP^K reaches the minimum of 0.31 at a 35 nm distance from the center of the GNP. Finally, we

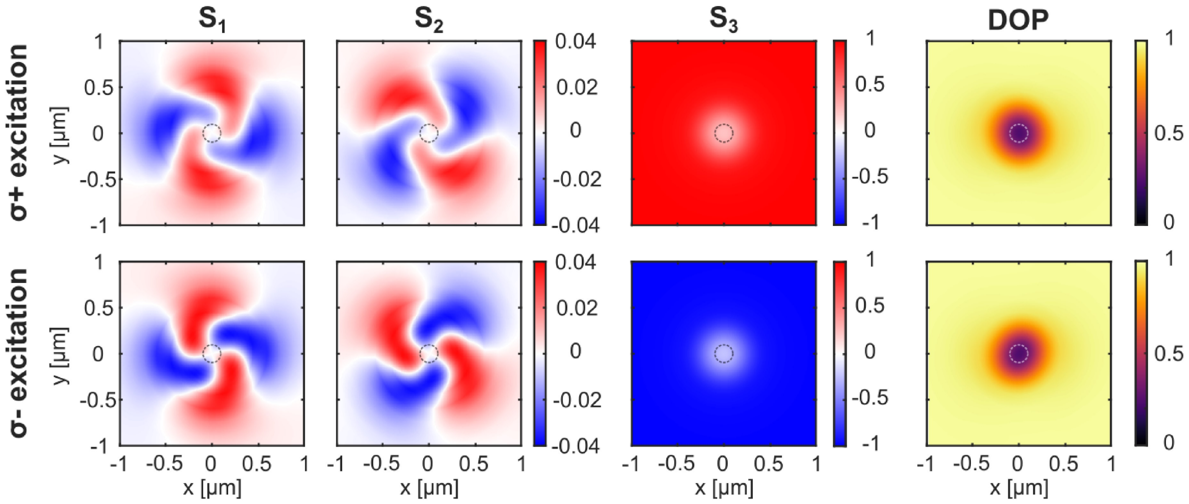

Fig. S8: Calculated Stokes parameters and DOP. Numerically computed spatial variation of Stokes parameters and the resulting degree of polarization in the vicinity of a GNP for σ^+ and σ^- excitation polarization. Here, the scanning measurement scheme is considered. The dotted circle denotes the projected GNP edge.

address the excitation aspect. In the main text, we derived the valley exciton densities $n_K(x_\mathrm{b}, x, y)$ for a focused Gaussian beam displaced along the x-axis. These densities can be generalized for an arbitrary beam position $(x_\mathrm{b}, y_\mathrm{b})$ by rotating the coordinate system at an

angle $\phi_\mathrm{b} = \mathrm{atan2}(y_\mathrm{b}, x_\mathrm{b})$ as follows

$$n_K^{2\mathrm{D}}(x_\mathrm{b}, y_\mathrm{b}, x, y) = n_K\left(\sqrt{x_\mathrm{b}^2 + y_\mathrm{b}^2}, x', y'\right), \text{ where } \begin{cases} x' = x\cos\phi_\mathrm{b} + y\sin\phi_\mathrm{b}, \\ y' = -x\sin\phi_\mathrm{b} + y\cos\phi_\mathrm{b}. \end{cases} \tag{8}$$

Now we have everything at hand to compute the farfield PL intensities $\tilde{\mathcal{I}}_{0/90}(x, y)$ and $\tilde{\mathcal{I}}_{45/135}(x, y)$ using an equation similar to equation (4) from the Methods section

$$\tilde{\mathcal{I}}_\alpha^{2\mathrm{D}}(x_\mathrm{b}, y_\mathrm{b}) \propto \iint \left[n_K^{2\mathrm{D}}(x_\mathrm{b}, y_\mathrm{b}, \xi, \eta) \cdot \mathcal{I}_\alpha^{2\mathrm{D},K}(\xi, \eta) + n_{K'}^{2\mathrm{D}}(x_\mathrm{b}, y_\mathrm{b}, \xi, \eta) \cdot \mathcal{I}_\alpha^{2\mathrm{D},K'}(\xi, \eta)\right] \mathrm{d}\xi\mathrm{d}\eta \tag{9}$$

with $\alpha = 0, 90, 45, 135$. These are used to compute the final $S_1(x, y)$ and $S_2(x, y)$. The spatially-resolved Stokes parameters and resulting DOP are shown for both excitation polarizations, σ^+ (top) and σ^- (bottom), in Figure S8. Note, that here we neglected non-zero intervalley scattering. Due to finite optical resolution and mixing of K and K' emitters, S_1 and S_2 do not exceed the value of 0.04, consistent with measurements shown in section S2. This result supports our conclusion that the reduction in DOP around the nanoparticle is mainly attributed to the decrease in DOCP.

S.8 Scanning electron microscope imaging of deposited gold nanoparticles

We have studied the size, shape and distribution of gold nanoparticles after deposition by means of scanning electron microscope (SEM) imaging. For this, we have deposited GNPs on a separate glass substrate coated with 15 nm of indium tin oxide as conductive layer to prevent charging of the sample during the imaging. Figure S9 (left) shows the respective top-view SEM image of a typical sample region. Our deposition process results in a homogeneous distribution of mostly isolated GNPs with few clusters of two or more GNPs. By locating

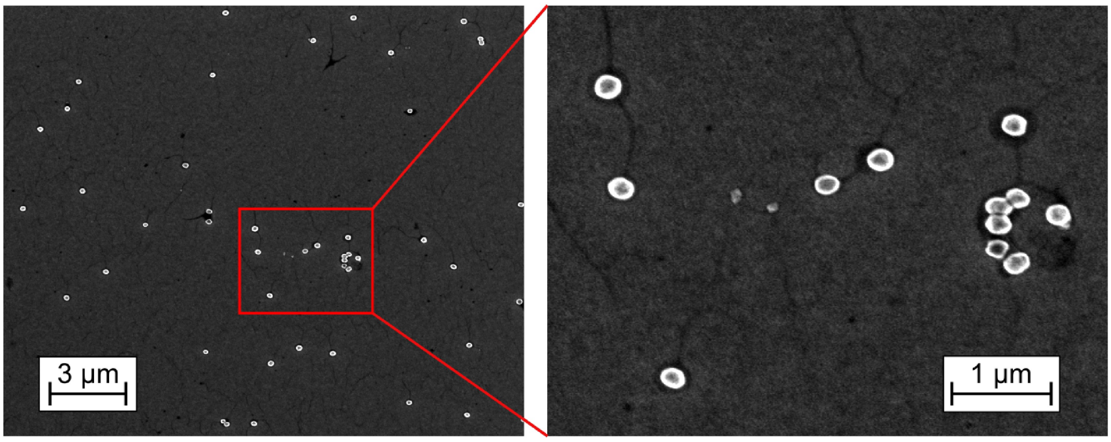

Fig. S9: Scanning electron microscope imaging. Top-view scanning electron microscope image of gold nanoparticles deposited onto a glass substrate coated with indium tin oxide. A zoomed in image of the region indicated by the red box is shown on the right side.

1168 individual GNPs using a circle detection algorithm, we have obtained an average GNP size of (220 ± 15) nm. On the right side we show a zoomed in image of the region indicated by the red box in order to identify the shape of individual GNPs. Slight deviations due to a finite ellipticity can occur for individual GNPs. Generally, elliptical nanoparticles can introduce a finite degree of linear polarization of the PL from emitters in their vicinity. However, in our PL measurements, we did not notice any significant enhancement of the S1 or S2 Stokes parameter at the position of the GNPs. Hence, the GNPs can be approximated as spherical with reasonable accuracy. Note that clusters of more than one GNP can also readily be distinguished from isolated GNPs by optical darkfield microscopy due to their different shape. Similarly, GNPs of significantly different sizes can be distinguished in darkfield microscopy by their different color impression due to the spectral shift of the plasmonic resonance. This allowed us to limit our PL measurements to 1L-MoS_2 decorated only with isolated GNPs with similar sizes.

References

(1) Collett, E. Field guide to polarization. 2005.

(2) Gramatikov, B. I. A Mueller matrix approach to flat gold mirror analysis and polarization balancing for use in retinal birefringence scanning systems. *Optik* **2020**, *207*, 164474.

(3) Fernandez-Corbaton, I.; Molina-Terriza, G. Role of duality symmetry in transformation optics. *Phys. Rev. B* **2013**, *88*, 085111.

(4) Zambrana-Puyalto, X.; Bonod, N. Tailoring the chirality of light emission with spherical Si-based antennas. *Nanoscale* **2016**, *8*, 10441–10452.

(5) Eismann, J. S.; Neugebauer, M.; Banzer, P. Exciting a chiral dipole moment in an achiral nanostructure. *Optica* **2018**, *5*, 954–959.
